# Beneficial Effects of Reconstituted High-Density Lipoprotein (rHDL) on Circulating CD34^+^ Cells in Patients after an Acute Coronary Syndrome

**DOI:** 10.1371/journal.pone.0168448

**Published:** 2017-01-06

**Authors:** Catherine Gebhard, Eric Rhéaume, Colin Berry, Geneviève Brand, Anne-Elen Kernaleguen, Gabriel Théberge-Julien, Mohammad Afaque Alam, Candace Y. W. Lee, Laurianne Boileau, Malorie Chabot-Blanchet, Marie-Claude Guertin, Marc-André Lavoie, Jean Grégoire, Réda Ibrahim, Philippe L'Allier, Jean-Claude Tardif

**Affiliations:** 1 Montreal Heart Institute, Montreal, Quebec, Canada; 2 Université de Montréal, Montreal, Quebec, Canada; 3 Institute of Cardiovascular and Medical Sciences, University of Glasgow, Glasgow, United Kingdom; 4 Montreal Heart Institute Coordinating Centre (MHICC), Montreal, Quebec, Canada; Centro Cardiologico Monzino, ITALY

## Abstract

**Background:**

High-density lipoproteins (HDL) favorably affect endothelial progenitor cells (EPC). Circulating progenitor cell level and function are impaired in patients with acute coronary syndrome (ACS). This study investigates the short-term effects of reconstituted HDL (rHDL) on circulating progenitor cells in patients with ACS.

**Methods and Findings:**

The study population consisted of 33 patients with recent ACS: 20 patients from the ERASE trial (randomized to receive 4 weekly intravenous infusions of CSL-111 40 mg/kg or placebo) and 13 additional patients recruited as controls using the same enrolment criteria. Blood was collected from 16 rHDL (CSL-111)-treated patients and 17 controls at baseline and at 6–7 weeks (i.e. 2–3 weeks after the fourth infusion of CSL-111 in ERASE). CD34^+^ and CD34^+^/kinase insert domain receptor (KDR^+^) progenitor cell counts were analyzed by flow cytometry. We found preserved CD34^+^ cell counts in CSL-111-treated subjects at follow-up (change of 1.6%), while the number of CD34^+^ cells was reduced (-32.9%) in controls (p = 0.017 between groups). The level of circulating SDF-1 (stromal cell-derived factor-1), a chemokine involved in progenitor cell recruitment, increased significantly (change of 21.5%) in controls, while it remained unchanged in CSL-111-treated patients (p = 0.031 between groups). *In vitro* exposure to CSL-111 of early EPC isolated from healthy volunteers significantly increased CD34^+^ cells, reduced early EPC apoptosis and enhanced their migration capacity towards SDF-1.

**Conclusions:**

The relative increase in circulating CD34^+^ cells and the low SDF-1 levels observed following rHDL infusions in ACS patients point towards a role of rHDL in cardiovascular repair mechanisms.

## Introduction

Several studies have consistently supported high-density lipoprotein (HDL)-cholesterol as a significant, strong, and independent inverse predictor of cardiovascular risk, even when low-density lipoprotein cholesterol (LDL-C) is reduced to very low levels by high dose statins[1]. While the inverse association between HDL-C and cardiovascular outcomes has been proven to be very robust, recent high profile pharmacological intervention studies and a Mendelian randomization analysis have challenged the concept that raising endogenous plasma HDL-C will uniformly translate into improved cardiovascular outcomes[2,3]. These recent studies have caused growing awareness that the effects of HDL may vary in different clinical settings and that an increase of dysfunctional HDL particles could also be detrimental, a phenomenon referred as ‘HDL dysfunction’. Indeed, population-based studies indicate that a substantial proportion of patients with ACS present with reduced or dysfunctional HDL which, in turn, is associated with a higher risk of early recurrent cardiovascular events[4,5,6]. As a consequence, exogenous HDL has been suggested as a treatment option for modifying the high-risk state following ACS and beneficial effects on coronary atherosclerosis in patients with ACS have been suggested after infusions of reconstituted HDL (rHDL)[7,8].

While the anti-atherosclerotic action of HDL is believed to be mostly related to its role in reverse cholesterol transport, experimental data indicate that rHDL may promote re-endothelialization by improving endothelial progenitor cell (EPC) levels and functionality[9]. Accordingly, low plasma HDL-C levels have been reported to be associated with a decreased number of EPCs[10]. Progenitor cell based therapies might also reduce short- and long-term recurrent cardiovascular events in patients with ACS[11], and *in vivo* data indicate that vascular repair by EPCs might be one of the underlying mechanisms[12,13]. Following percutaneous coronary intervention (PCI), bone marrow-derived stem and vascular progenitor cells that express stem-cell-like antigens such as CD34 are mobilized, rapidly recruited to sites of injury thereby inhibiting further platelet activation and leading to neovascularization, improved left ventricular function and reduced myocardial lesion area[14,15]. However, several populations, including patients with ACS, seem to fail to respond to PCI with progenitor cell mobilization, resulting in increased mortality and more significant left ventricular remodeling[16,17,18,19].

An epidemiologic study showed an association of statin use with higher CD34^+^ progenitor cell counts, thereby supporting the hypothesis that levels of EPCs may be influenced therapeutically[20]. Indeed, moderate-dose atorvastatin increased CD34^+^ cells in patients with myocardial infarction, and systemic rHDL infusion can improve the availability of CD34^+^ cells in patients with type 2 diabetes[21]. However, whether infusions of rHDL can favorably influence EPCs or CD34^+^ progenitor cells in the setting of recent ACS is not known.

Given that 1- endogenous HDL and progenitor cell functions are impaired in ACS patients, a population characterized by a high short-term risk for recurrent ischaemic events, 2- EPCs, CD34^+^ progenitors and rHDL may exert rapid beneficial effects on some atherosclerotic plaque characteristics, and 3- rHDL increases EPC levels *in vivo* in patients with diabetes, we hypothesized that some of the beneficial effects of rHDL infusions may be mediated via an improvement of circulating EPC or CD34^+^ progenitor levels and function in patients with ACS.

## Methods

### Subjects

The study population consisted of 33 patients with recent ACS: 20 patients from the ERASE trial (16 CSL-111-treated and 4 placebo-treated patients) and 13 additional patients who were recruited as controls using the ERASE enrolment criteria[8]. Further, twenty-six patients without ACS and with normal coronary arteries who underwent coronary angiography for different reasons served as controls for baseline EPC measurements. Details of the ERASE trial were previously published[8]. Briefly, ERASE was a randomized, double-blind, placebo-controlled, multicentre trial which evaluated the effects of the rHDL CSL-111 (CSL Ltd, Parkville, Australia) on plaque burden as assessed by intravascular ultrasonography in patients who were recruited within 2 weeks of an ACS, defined as unstable angina, non-ST-segment elevation myocardial infarction (MI) or ST-segment elevation MI. Patients with significant left main coronary artery disease (≥ 50% stenosis), renal insufficiency, liver disease, active cholecystitis, uncontrolled diabetes mellitus, New York Heart Association (NYHA) class III or IV heart failure, known soybean allergy, history of alcohol or drug abuse, planned anticoagulation treatment, or previous or planned coronary bypass graft surgery were excluded from study participation. Patients were randomized to receive 4 weekly intravenous infusions of placebo or CSL-111 40 mg/kg. Blood collection was performed at baseline (prior to the first infusion) and then at 6 to 7 weeks (2 to 3 weeks after the fourth study infusion). For the 13 additional control subjects with ACS recruited, blood was also collected at baseline and at 6 to 7 weeks (similar to the subjects of the ERASE trial).

To evaluate the effects of CSL-111 on cell adhesion, growth, apoptosis and migration, we performed *in vitro* experiments on blood collected from 10 additional healthy subjects. The study complied with the declaration of Helsinki and was approved by the Institutional Review Board of the Montreal Heart Institute, with all subjects providing written informed consent.

### Blood Sampling and Circulating Progenitor Quantification by Flow Cytometry

Venous blood was collected in the recumbent position (35 mL in potassium-EDTA-containing tubes and 5 mL in tubes without anticoagulant for separation of serum). The blood samples were immediately transported to laboratories for processing. EPCs were quantified in triplicate using previously reported guidelines for progenitor cell enumeration[22] with some modifications. Briefly, 100 μL of blood was immunostained for 10 minutes at room temperature with anti-human KDR antibody[23,24] (Abcam, Cambridge, MA; conjugated using Zenon® Alexa Fluor®647-RPE labeling kit, Invitrogen, Burlington, ON). Then, Stem-Kit™ (Beckman Coulter, Brea, CA) monoclonal antibodies (mAbs) were added using a fluorescein isothiocyanate (FITC)-conjugated anti-human CD45 antibody and a phycoerythrin (PE)-conjugated anti-CD34 antibody for 20 minutes at room temperature. Isotype-identical antibodies were used as controls. Following incubation, erythrocytes were lysed using 1x NH_4_Cl lysing solution provided with the Stem-Kit for 10 minutes at room temperature prior to cytometry analysis. Stem-count fluorospheres were added to samples to determine absolute EPC counts by flow cytometry (Beckman Coulter EPICS® XL™ flow cytometer).

**Fig 1:**
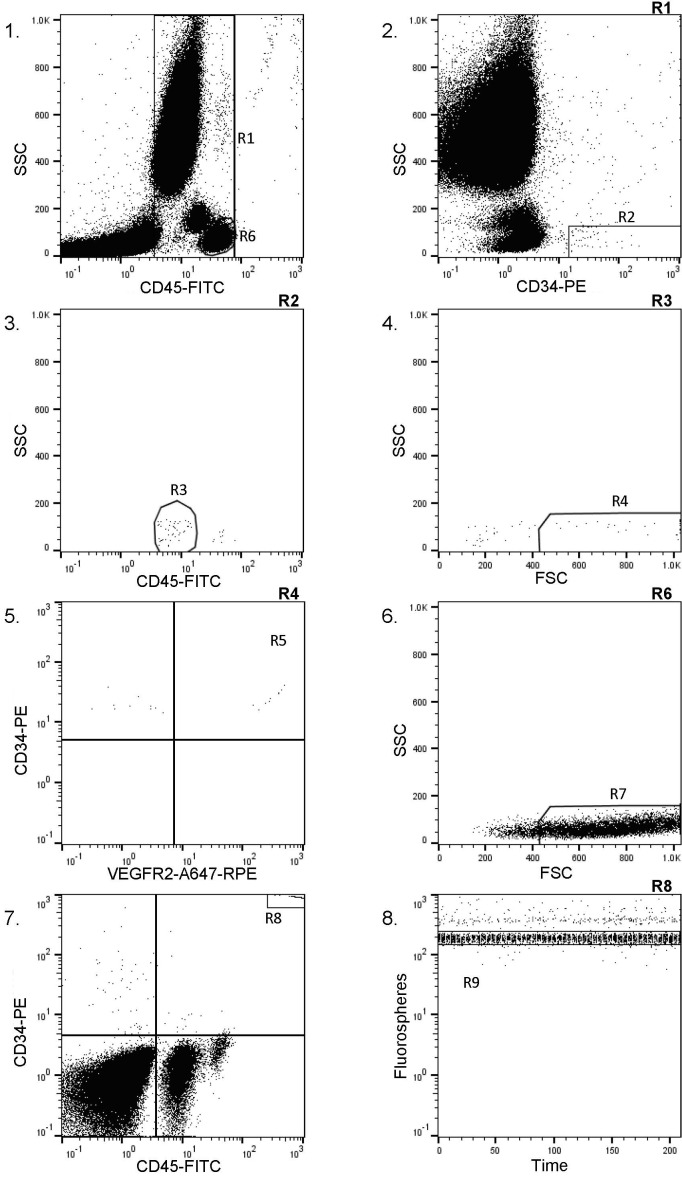
Representative example of sequential gating strategy for flow cytometric analysis of endothelial progenitor cells. A modified ISHAGE strategy was applied for EPC quantification. 1) Representative sample stained with CD45-FITC. Region R6 represents lymphocytes. 2) Anti-CD34-PE staining of cells from R1. Region R2 represents CD34^+^ cells. 3) Region R3 is placed to include the low Side Scatter and low to intermediate CD45 staining. 4) R4 represents all events from regions R1, R2 and R3 displayed on a FSC vs SSC dot plot to confirm that the selected events fall into a lymph-blast region. 5) Displays the events included in regions R1, R2, R3 and R4. A quadrant was positioned to separate the positive and the negative cells for VEGFR2 staining. An appropriate isotype control was used to adequately place the quadrant. Region R5 represents the total EPCs (CD34+/VEGFR2+ cells). 6) Events from region R6. This region is used to set the region R4. 7) All events. This histogram is useful to establish the lower limit of CD45 expression for the CD34^+^ events. The region R8 is placed in the right top of the histogram to count all Stem-count fluorospheres accumulated for each sample for absolute quantification. 8) Events from region R8. This region includes the Stem-count fluorospheres singlet population.

We used a modification of the Stem-Kit protocol which itself is based on the ISHAGE guidelines for CD34^+^ cell determination by flow cytometry[25]. As indicated in a representative example (Fig 1), histogram 1 displays all events except the fluorospheres (shown on histogram 7, R8). The region R1 is positioned to include all CD45^+^ events. This region will exclude CD45 negative events (i.e. red blood cells, platelets and cell debris). The region R6 represents lymphocytes (bright CD45, low scatter). Histogram 2 displays events from region R1. The region R2 is adjusted to include CD34^+^ cells with low Side Scatter. Histogram 3 is showing the events from regions R1 and R2. The region R3 is placed to include the low Side Scatter and low to intermediate CD45 staining. Histogram 4 represents all events from regions R1, R2 and R3 displayed on a FSC vs SSC dot plot to confirm that the selected events fall into a lymph-blast region (R4). CD34^+^ cells number is counted in the region R4 (events meeting all the fluorescence and light scatter criteria of ISHAGE Guidelines for CD34^+^ cells). CD34^+^ number determination was performed in triplicate for each patients and the mean CD34^+^ value was used. An appropriate isotype control was used as a control. Histogram 5 displays the events included in regions R1, R2, R3 and R4. A quadrant was positioned to separate the positive and the negative cells for VEGFR2 staining. An appropriate isotype control was used to adequately place the quadrant. Region R5 represents the total EPCs (CD34^+^/VEGFR2^+^ cells). Histogram 6 shows events from region R6. This region is used to set the region R4 (histogram 4) to include events no smaller than lymphocytes. Histogram 7 represents all events. This histogram is useful to establish the lower limit of CD45 expression for the CD34^+^ events. The region R8 is placed in the right top of the histogram to count all Stem-count fluorospheres accumulated for each sample for absolute quantification. Histogram 8 shows events from region R8. This region includes the Stem-count fluorospheres singlet population. It is used to verify that fluorospheres accumulate constantly over time. Absolute numbers of CD34^+^ and CD34^+^/KDR^+^ cells per μL of blood were determined and results were expressed as relative changes from the respective baseline values.

### ELISA Assays

Serum levels of VEGF and stromal cell-derived factor-1 (SDF-1) were quantified by ELISA (Quantikine kits, R&D Systems).

### *In Vitro* Experiments with CSL-111

One hundred mL of venous blood was collected on sodium citrate anticoagulant from each of the 7 healthy volunteers for *in vitro* experiments (exposure of early EPC (eEPC) to CSL-111). Peripheral blood mononuclear cells (PBMCs) were isolated by density gradient centrifugation (Ficoll-Paque™ PLUS medium, GE Healthcare, QC), plated at a density of 1.5 x 10^6^/cm^2^ on four fibronectin-coated plates (BD Biosciences, Mississauga, ON), and cultured under 5% CO_2_ (37°C) in endothelial growth media (EGM®-2, Lonza, QC), which was supplemented with 20% embryonic stem cell-qualified fetal-bovine serum (Invitrogen, Burlington, ON). For each of the dishes, cells were exposed to 1 mg/mL of CSL-111 for either days 0 to 4, days 4 to 7, days 0 to 7, or unexposed. Non-adherent cells were removed by washing after 4 days in culture, for eEPC culture. On day 7, adherent cells were harvested and stained with antibodies against CD34 (FITC-conjugated) and KDR (APC-conjugated). Cells were incubated for 30 minutes at 4°C before the addition of 50 000 SpheroTM AccuCount fluorescent beads (Spherotech Inc.) and their analysis on a BD LSRII flow cytometer. Apoptosis of the treated and untreated eEPCs was quantified in the following fashion: After 7 days in culture, eEPCs were washed, harvested by mild treatment with dispase (0.5 mg/mL), stained with Annexin V conjugated to Alexa Fluor 350 and with propidium iodide (BD Biosciences, Mississauga, ON) and then analyzed by flow cytometry.

### Transwell Migration Assay

eEPC migration was assessed using modified Boyden chambers. Briefly, eEPCs from healthy volunteers (n = 3) were cultured in presence or absence of CSL-111 as above and then harvested and resuspended in DMEM containing 10% (v/v) FBS. A polycarbonate filter membrane (5-μm-pore-diameter; Neuroprobe) was placed on the top of the lower wells, the latter filled with the medium mentioned above and supplemented with SDF-1 (100 ng/mL) as a chemoattractant. The chamber was tightened and cell suspensions (4 x 10^5^ cells/cm^2^) were added to the upper wells. After allowing cell migration for 16 hours, cells were scraped from the upper side of membranes using Kimwipes/cotton swab. The membrane with migrated cells was fixed and stained using Diff-quick (Thermo Fisher Scientific) staining kit. Stained cells were then counted directly under the microscope using 40X objective. Each experiment was assayed in quadruplicate and 3 randomly selected high-power fields for each well were counted to determine the number of cells that had migrated. Migration is presented as the chemotactic index, obtained by division of the number of migrating cells in the treated groups by the number of migrating cells in the corresponding control wells.

### Statistical Analyses

Statistical analyses were conducted at the Montreal Heart Institute Coordinating Centre (MHICC) using SAS (version 9.1 or higher, SAS Institute, Cary, NC). Categorical data were expressed as frequencies and percentages. For continuous variables, depending on the distribution of the data, results are expressed as mean ± standard deviation or median (Q1;Q3). Baseline characteristics were compared between groups using Student t-test or chi-square test where appropriate. Comparisons of experimental data between the CSL-111-treated group *vs* control were made by the Mann-Whitney test. Between-group comparisons of the time lapses between ACS and baseline blood collection as well as between baseline and follow-up were assessed using the Student t-test or the Mann-Whitney test, as appropriate. For the parameters measured during the CSL-111 *in vitro* treatments, the overall effect of exposure to CSL-111 (days 0 to 4, days 4 to 7, days 0 to 7, or unexposed) was tested using the Friedman test and, if significant, Wilcoxon signed-rank tests were used to compare paired types of exposure. A mixed model analysis of variance (ANOVA) with terms for block (to account for the fact that patients contribute to data in quadruplicate), exposure to CSL-111, SDF-1 (yes/no) and interaction exposure to CSL-111 x SDF-1 was used to compare the EPC among the combinations of exposure to CSL-111/SDF-1 in the migration assay. The appropriate pairwise comparisons followed if global F tests were significant. Statistical significance was defined as p < 0.05.

## Results

The baseline characteristics of the groups of CSL-111-treated patients and control patients were similar (Table 1). Also, there was no significant difference between groups in terms of days from presentation with ACS to the time of baseline blood collection for progenitor cells (3.3+/-2.8 and 2.6+/-1.4 days after myocardial infarction (MI) for controls and CSL-111-treated patients, respectively) and in the median number of days from ACS event to the follow-up sampling (43 [40–46] and 39 [39–41] days after MI for controls and CSL-111-treated patients, respectively). A slightly higher BMI was found in the 4 placebo-treated patients as compared to the 13 additional patients who were recruited as controls using the ERASE enrolment criteria (p = 0.04).

**Table 1:** Baseline characteristics of the subjects.

	Control (n = 17)	CSL-111 (n = 16)
**Age (years)**	**55±11**	**57±9**
**Male (n (%))**	**14 (82)**	**16 (100)**
**Weight (kg)**	**86.5±22.4**	**90.3±17.5**
**BMI (kg/m**^**2**^**)**	**29.4±6.5**	**29.2±5.0**
**Diabetes (n (%))**	**2 (12)**	**1 (6)**
**Hypertension (n (%))**	**11 (65)**	**11 (69)**
**Current tobacco use (n (%))**	**6 (35)**	**4 (25)**
**Total cholesterol (mmol/L)**	**4.7±1.4**	**4.6±1.0**
**LDL cholesterol (mmol/L)**	**2.7±1.1**	**2.6±0.9**
**HDL cholesterol (mmol/L)**	**1.2±0.3**	**1.2±0.3**
**Triglycerides (mmol/L)**	**1.9±0.9**	**1.8±0.9**
**Use of lipid lowering medication (n (%))**	**15 (88)**	**15 (94)**
**Use of inhibitors of the renin-angiotensin system (n (%))**	**12 (71)**	**11 (69)**
**Prior PCI (n (%))**	**10 (59)**	**13 (81)**
**Unstable Angina (n (%))**	**13 (76)**	**12 (75)**
**NSTEMI (n (%))**	**2 (12)**	**3 (19)**
**STEMI (n (%))**	**2 (12)**	**1 (6)**

Values shown are mean±SD for continuous variables or frequencies and percentages for categorical variables. No statistically significant difference between groups for all the parameters listed. BMI, body mass index; HDL, high-density lipoprotein; LDL, low-density lipoprotein; PCI, percutaneous coronary intervention; NSTEMI, non ST-elevation myocardial infarction; STEMI, ST-elevation myocardial infarction.

**Fig 2:**
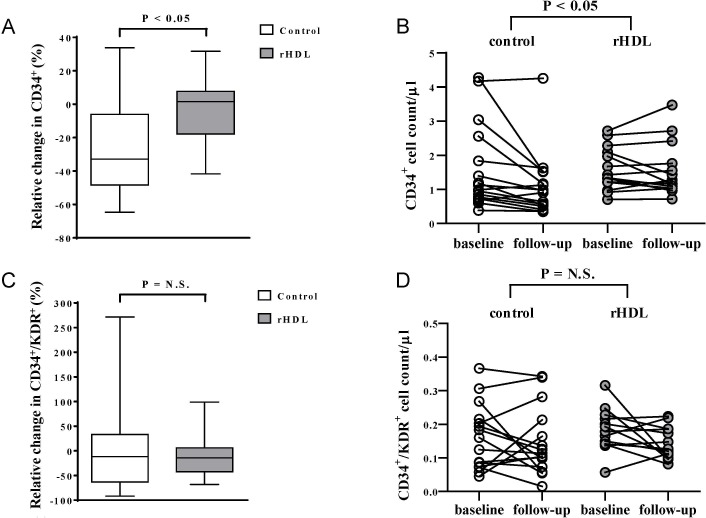
Relative preservation of CD34^+^ cells in patients with acute coronary syndrome following treatment with reconstituted high-density lipoprotein (rHDL) compared to controls. The CD34^+^ progenitor cells (A and B) and CD34^+^/KDR^+^ endothelial progenitor cells (C and D) were quantified in blood samples collected at baseline and at follow-up. The follow-up samples for the CSL-111-treated group were obtained 16 ± 4 days following completion of the 4 weekly rHDL infusions. Each box plot in A and C shows the median, the interquartile range, the maximum and the minimum of the relative change. B and D show absolute numbers of CD34^+^ (B) and CD34^+^/KDR^+^ (D) endothelial progenitor cell count at baseline and at follow-up. p < 0.05 between groups (A and B), p = N.S. (C and D) from Mann-Whitney tests.* one outlier (baseline cell count 1.6/μl, follow-up 0.1/μl) not presented in figure due to axis limits.

We first measured the levels of circulating progenitor cells in ACS patients and normal controls at baseline. As compared to patients with normal coronary arteries (n = 26), patients with ACS (n = 29) had significantly higher levels of both, CD34^+^ and CD34^+^/KDR^+^ progenitor cells (p<0.0001 and p = 0.001, respectively, Figure A in S1 File, S1 Table). The follow-up samples for the CSL-111-treated ACS patients were obtained 16 ± 4 days following completion of the 4 weekly rHDL infusions. The median relative changes in CD34^+^ progenitor cells, as quantified by flow cytometry from blood samples collected at baseline and in the follow-up, were -32.9% and 1.6% for the control group and CSL-111-treated group, respectively (p = 0.017, Fig 2A, S2 Table). These significant differences between control group and CSL-111-treated group persisted when changes in CD34^+^ progenitor cells in relation to total number of leucocytes (CD45^+^ cells) were analysed (p = 0.03, Figure B in S1 File). In contrast, the median relative changes in CD34^+^/KDR^+^ endothelial progenitor cells were -11.7% (control group, n = 16) and -14.2% (CSL-111, n = 13) without any significant difference between groups (p = 0.98, Fig 2C, S2 Table). Similarly, no changes in total peripheral leucocyte count (CD45^+^) were observed following rHDL treatment as compared to controls (Figure B in S1 File). Changes of absolute CD34^+^ and CD34^+^/KDR^+^ endothelial progenitor cell count before and after treatment are indicated in Fig 2B, 2D, and S2 Table.

**Fig 3:**
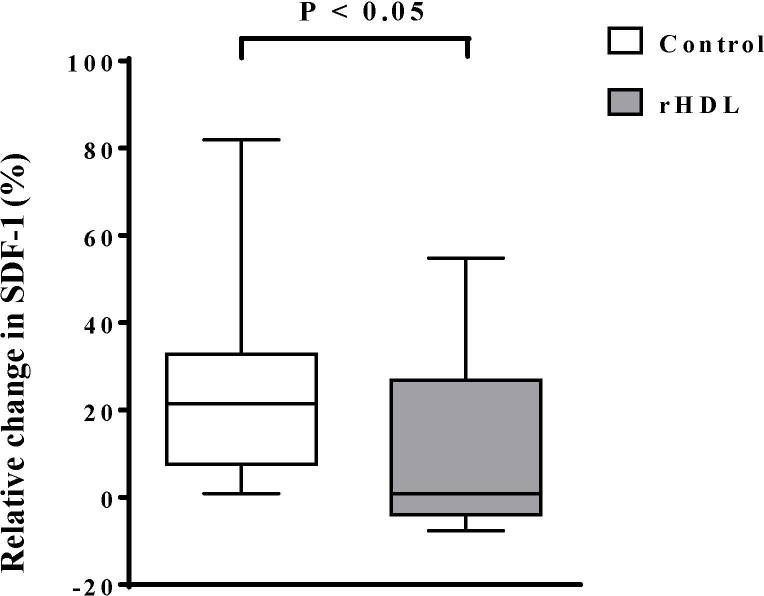
Decreased levels of serum stromal cell-derived factor-1 (SDF-1) in patients with acute coronary syndrome following treatment with reconstituted high-density lipoprotein (rHDL) compared to controls. Relative changes from baseline in serum SDF-1 in the control group and in the rHDL-treated group. Each box plot shows the median, the interquartile range, the maximum and the minimum. p < 0.05 between groups from Mann-Whitney test.

We next investigated whether the serum concentrations of cytokines known to be involved in the mobilisation of vascular progenitor cells such as VEGF and SDF-1 were affected by the CSL-111 treatment. Although we did not observe differences between the groups in the serum concentrations of VEGF (data not shown), we found that the median relative change in serum SDF-1 from baseline was 21.5% in controls and 0.9% in the CSL-111-treated group (Fig 3, S3 Table, p = 0.031).

**Fig 4:**
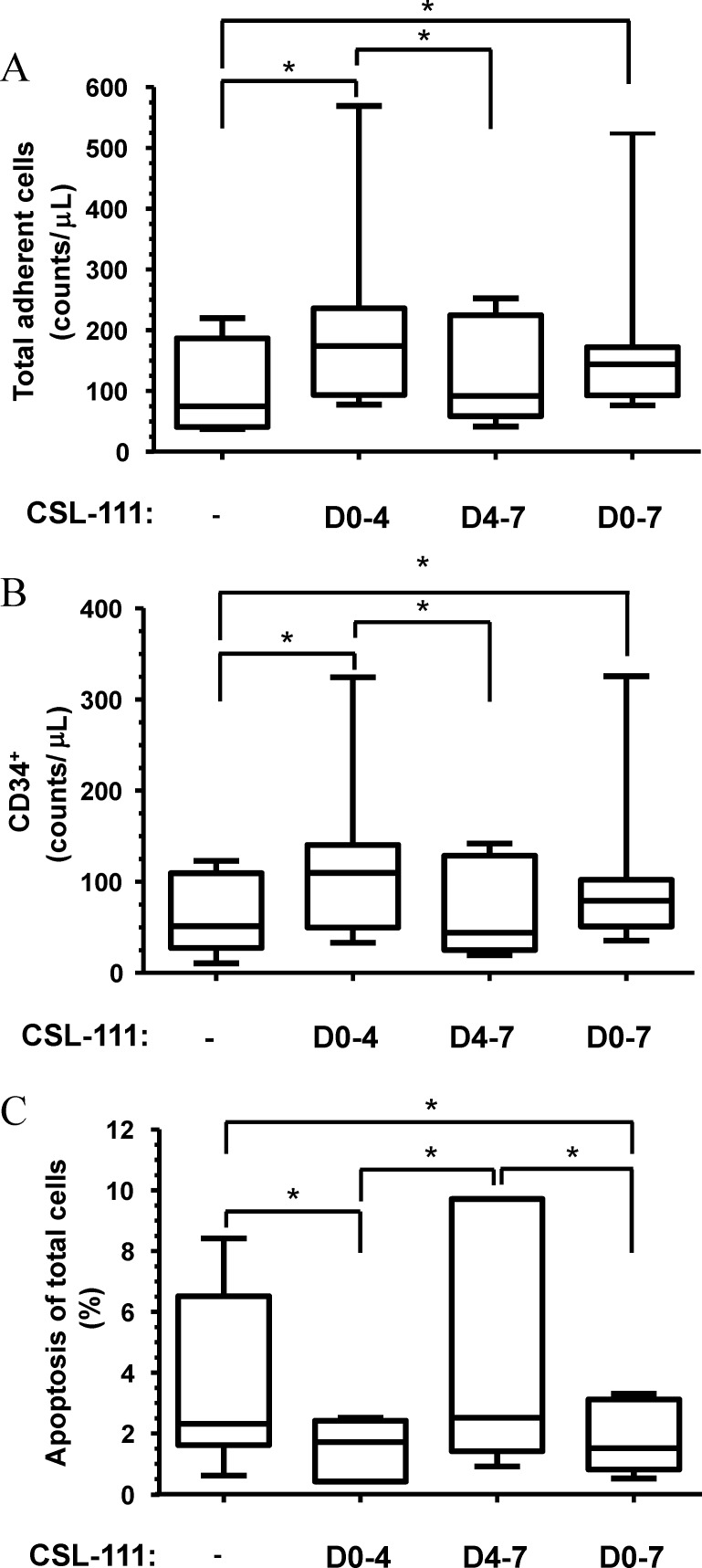
*In vitro* exposure of circulating progenitor cells to CSL-111. Peripheral blood mononuclear cells (PBMCs) were isolated from healthy donors (n = 7) and plated on fibronectin-coated plates in the absence or presence of CSL-111 (1 mg/mL) from day 0 to day 4 (D0-4), 4 to 7 (D4-7) or 0 to 7 (D0-7). After 7 days of culture, adherent cells were harvested and analyzed by flow cytometry. (A) All adherent cells were quantified by flow cytometry using cell counting beads for enumeration. (B) CSL-111 treatment increases the total number of CD34^+^ cells when added to cell culture media at D0-4 and D0-7; CD34^+^ cells were quantified by flow cytometry. (C) CSL-111 treatment reduces basal apoptosis in eEPCs when added to cell culture media at D0-4 and D0-7. Apoptosis was measured by flow cytometry using Annexin V labeling. Each box plot shows the median, the interquartile range, the maximum and the minimum of the relative change. * indicates p < 0.05 between groups from Wilcoxon signed-rank tests.

To further analyze the effects of CSL-111, we next isolated PBMCs from healthy subjects and assessed the effect of exposing them to CSL-111 during culture and differentiation of eEPC. Early EPCs were non-treated or treated with CSL-111 (1 mg/mL) either from day 0 to 7, day 0 to 4, or day 4 to 7. Day 4 is the day when non-adherent cells were washed out and media was changed in the procedure for eEPC culture. We obtained a median number of total fibronectin-adherent eEPC that was 1.9-fold higher when cells were incubated for 7 days in presence of CSL-111 than when cells were not exposed to CSL-111 (Fig 4A, S4 Table, p = 0.031). A similar increase in eEPC was seen when cells were incubated with CSL-111 from day 0 to 4 (p = 0.016 versus untreated cells) whereas eEPC exposure to CSL-111 from day 4 to 7 did not have a significant effect on the total number of eEPCs when compared to untreated cells. Similarly, we observed an increase in the median number of CD34^+^ eEPC when cells were exposed to CSL-111 during days 0 to 7 (1.5-fold higher) and 0 to 4 (2.1-fold higher) (Fig 4B, S4 Table, p-values 0.031 and 0.016, respectively). We also observed a decrease in the median values of the percentage of eEPC apoptosis when cells were treated with CSL-111 from days 0 to 7 or 0 to 4 compared to untreated cells (Fig 4C, S4 Table, p-values 0.016 for both treatment types).

**Fig 5:**
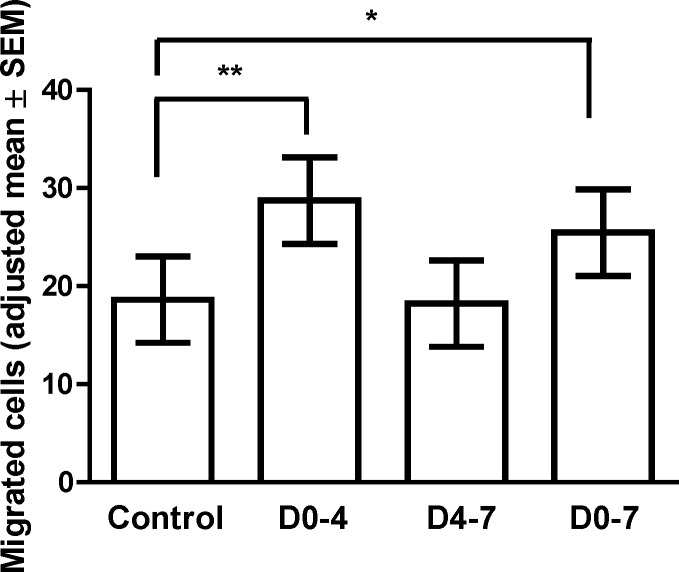
*In vitro* studies on the effect of CSL-111 on migratory capacity of eEPC. Peripheral blood mononuclear cells (PBMCs) were isolated from healthy donors (n = 3) and plated on fibronectin-coated plates in the absence or presence of CSL-111 (1 mg/mL) from day 0 to day 4 (D0-4), 4 to 7 (D4-7) or 0 to 7 (D0-7). On day 7 of culture, adherent cells were harvested and assayed in a modified Boyden chamber for their capacity to migrate along an SDF-1 gradient. Significantly increased migration was observed among cells treated with CSL-111 for day 0 to day 4 (**; p = 0.0003) and 0 to 7 (*; p = 0.0135) compared to controls. Figure shows adjusted mean±standard error of the mean (SEM). p-values are reported from mixed model ANOVA.

We also characterized the effect of CSL-111 on eEPC migration toward SDF-1 as a chemotactic agent. We observed that cells that were treated with CSL-111 during days 0 to 4 or 0 to 7 had a higher migration capacity toward SDF-1 (Fig 5, S5 Table, p = 0.0003 and 0.0135, respectively) compared to controls, whereas exposure of eEPC from day 4 to 7 did not result in an increase of migration capacity of the cells compared to control (not exposed to CSL-111) eEPCs. Western blot analysis of the expression of SDF-1 receptor CXCR4 indicates that CSL-111 increased protein expression of CXCR4 in eEPCs when it was present from day 0 to 4 or 0 to 7 (Figure C and Protocol A in S1 File), similar to migration data.

## Discussion

This is the first clinical study exploring short-term effects of intravenous rHDL infusions on circulating progenitor cell number and function in patients with recent ACS. In line with previous reports we observed that the number of circulating progenitor cells including CD34^+^ and CD34^+^/KDR^+^ cells increases acutely following an ACS[26,27]. An early decline of EPC levels or failure to mobilize EPCs from the bone marrow have been described in ACS patients, which in turn is associated with increased mortality[26,28,29]. We report here that the decline in progenitor cells can be prevented by four weekly infusions of rHDL (CSL-111), while a significant drop of 33% in CD34^+^ cell count is observed in untreated control patients.

Low progenitor cell numbers following PCI for ACS or stable coronary artery disease are associated with an increased risk of mortality and recurrent major adverse cardiac events (MACE), with the risk of MACE being highest during the first six months following the primary event[4,18,30]. Remarkably, changes in CD34^+^ progenitor counts in our study population were observed after only a short duration of treatment underlining the rapid action of rHDL on progenitor cell levels. Similarly, beneficial changes in some plaque characteristics were observed in the ERASE study after only four weeks of rHDL treatment[8]. Thus, the present study supports the hypothesis that HDL-raising strategies may exert at least part of their potentially beneficial effects on plaque morphology via an improvement of circulating bone marrow-derived progenitor levels[8].

One of the major limitations in studying EPCs is the lack of consensus on the identity of ‘true’ EPCs, which in turn limits the translation of EPC research into clinical studies. The surface marker profile of progenitor cells changes during the process of mobilization and maturation; as they mature, EPCs, a subtype of peripheral blood monocytes that express stem-cell-like antigens such as CD34, lose the CD133 marker and acquire vascular endothelial growth factor (VEGF) receptor-2, also known as KDR[31]. Thus, CD34^+^ cells form a more generic population of ‘early’ progenitor cells, while CD34^+^KDR^+^ cells are committed to the endothelial lineage[32]. In our study, we detected preserved numbers of CD34^+^ cells in patients receiving rHDL infusions, while there was no significant difference in CD34^+^/KDR^+^ cells in patients treated with rHDL compared to controls. However, there is conflicting evidence regarding the predictive power of CD34^+^/KDR^+^ cells in patients with CAD; while some studies have shown that the number of circulating CD34^+^/KDR^+^ cells predicts outcome in healthy individuals and patients with CAD[33,34], a comparative analysis in patients with ACS reported that the CD34^+^/CD133^+^ phenotype, but not the CD34^+^/KDR^+^ or the CD133^+^/KDR^+^ phenotype, is predictive of recurrent ACS or MACE[35]. A pooled analysis from four longitudinal studies, however, demonstrated that both CD34^+^ cells and CDR34^+^/KDR^+^ cells showed consistent results, suggesting that there is no clear evidence that one phenotype is superior to the other in terms of risk prediction[30]. These apparent discrepancies could be attributed to the very low number of CD34^+^/KDR^+^ EPCs in blood samples and the consequential high interobserver variability in assessing their quantity, the different methods and time points used to assess EPC in humans, and the relatively small number of patients in heterogeneous populations assessed[36]. Furthermore, previous data suggest that CD34^+^ cell level is more stable over time than CD34^+^/KDR^+^ cell level, which may be more influenced by pharmacological treatment[30,37]. Indeed, in patients with ACS, a reduced number of CD34^+^ rather than CD34^+^/KDR^+^ EPCs has been shown to be predictive of recurrent ACS[35,38,39,40]. In light of these studies and our results, one might conclude that CD34^+^ cells play an important role in the vascular repair process, particularly in the setting of an acute ischemic event. However, the mechanisms explaining how rHDL exerts different effects on the two progenitor populations are unclear and await further studies.

It has been shown previously that the percentage of apoptotic CD34^+^ progenitor cells is significantly increased in patients with ACS as compared to healthy subjects and is associated with the extent of coronary stenosis by angiography[41]. Thus, functional impairment of progenitor cells through enhanced apoptosis may underlie atherogenesis and cardiovascular events, while improving survival seems to be vital for neovascularization and arterial injury repair[42]. In our study, additional experiments using eEPCs isolated from healthy subjects demonstrated favourable effects of early administration of rHDL on eEPC apoptosis. These findings are consistent with the known anti-apoptotic effects of rHDL in another setting[43] and raise the possibility that early administration would be relevant for maximizing the therapeutic benefits of rHDL infusions in ACS patients. We speculate that a reduction in apoptosis might be one possible mechanism for the relative preservation of CD34^+^ cell counts following rHDL infusions. The reduction in apoptosis, seen in our *in vitro* study, was paralleled by a higher migration capacity towards SDF-1 in eEPC cell cultures treated with CSL-111. A reduction in EPC migratory and proliferation capacity was previously observed in patients with ACS[44] and correlates with increased atherosclerotic load in humans[45]. Therefore, our findings with eEPCs raise the hypothesis that an increase in progenitor cells level and migration capability could contribute to favourable effects of rHDL in patients with ACS.

We also measured the circulating concentration of chemokines known to be involved in EPC recruitment, such as stromal cell-derived factor-1 (SDF-1; also known as CXCL12) and vascular endothelial growth factor (VEGF). We observed that CSL-111-treated patients had lower SDF-1 circulating levels than controls; however, this finding is counterbalanced by our observation of enhanced SDF-1-mediated migration following CSL-111 treatment *in vitro*. Further, there were no differences in VEGF levels between the treatment and control groups. This is in line with previous observations where no correlation between levels of SDF-1 or VEGF and numbers of vascular progenitors in patients with ACS were observed[14,16]. Indeed, chemokine levels have been shown to undergo rapid changes in experimental models where SDF-1 levels increase sharply 3 days post-MI but go back to normal levels after 1 week[46]. Thus, our findings could be explained by a reduction of SDF-1 levels that may have occurred through negative feedback-mechanism as a consequence of better cardiovascular tissue repair due to improved adhesion of progenitor cells to damaged tissues. Furthermore, the improved migration capacity observed in cultured eEPC following rHDL treatment may explain why patients treated with rHDL managed to maintain higher levels of CD34^+^ progenitor cells despite lower SDF-1 levels. Interestingly, experimental work suggests that SDF-1 signalling could even be detrimental for infarct size and left ventricular function in an ischemia–reperfusion injury model, due to the recruitment of inflammatory cells and fibrocytes[47]. Thus, based on these conflicting data and the high individual variability of SDF-1 levels observed in clinical studies, it has been proposed that for progenitor cell homing the local expression of SDF-1 in the heart is more important than SDF-1 blood levels[14]. Further studies are necessary to clarify and definitively assess the role of SDF-1 signalling in EPC mobilization during ischemia.

There are limitations to our study. Our finding of a potential beneficial effect of rHDL on progenitor levels and function is limited by a small sample size which led us to enrol additional control subjects. In addition, our small study population precluded further subgroup analyses pertaining to the metabolic syndrome and diabetes which are known to be associated with reduced EPC counts and function[21,48,49]. Prospective studies are required to specifically evaluate the therapeutic potential of HDL infusions in these subpopulations in the clinical setting of ACS.

In conclusion, we have found that in patients suffering from ACS, rHDL administration preserves circulating CD34^+^ levels, possibly via beneficial effects on improved migration and reduced apoptosis of progenitor cells. Prospective clinical trials are needed to evaluate whether CD34^+^ cell count may be a useful biomarker in the evaluation of novel HDL-raising therapies, particularly those where rHDL or mimetics are involved.

## Supporting Information

S1 File Supplementary Figures and Protocols. (DOCX)

S1 Table Endothelial progenitor cell count in patients with acute coronary syndrome (ACS) and controls. CD34^+^ (left) and CD34^+^/KDR^+^ (right) endothelial progenitor cell counts at baseline in patients with ACS and patients with normal coronary arteries (normal) as assessed by coronary angiography.
(PPTX)

S2 Table Endothelial progenitor cell count in patients with acute coronary syndrome. Left panel: CD34^+^ progenitor cells before and after treatment with reconstituted high-density lipoprotein (rHDL) compared to controls (Placebo treatment). Right panel: CD34^+^/KDR^+^ endothelial progenitor cells before and after treatment with reconstituted high-density lipoprotein (rHDL) compared to controls (Placebo treatment).
(PPTX)

S3 Table Serum stromal cell-derived factor-1 (SDF-1) in patients with acute coronary syndrome after treatment with reconstituted high-density lipoprotein (rHDL). (PPTX)

S4 Table In vitro exposure of peripheral blood mononuclear cells (PBMCs) to CSL-111. Total adherent (left), CD34^+^(middle) and apoptotic (right) cell count in the absence (control) or presence of CSL-111 (1 mg/mL). D, day.
(PPTX)

S5 Table Effect of CSL-111 on migratory capacity of endothelial progenitor cells derived from healthy donors. Chemotactic index of endothelial progenitor cells indicating their capacity to migrate along an SDF-1 gradient following treatment with CSL-111 for different periods of time.
(PPTX)
