# Supplementary material for: Beneficial Effects of Reconstituted High-Density Lipoprotein (rHDL) on Circulating CD34+ Cells in Patients after an Acute Coronary Syndrome
Source: PLoS One. 2017 Jan 6;12(1):e0168448. doi: 10.1371/journal.pone.0168448 (PMC5218493; doi:10.1371/journal.pone.0168448)
Supplement: S1 File — (DOCX) [file pone.0168448.s001.docx]

**Figure A in S1 File.** CD34^+^ (Left) and CD34^+^/KDR^+^ (Right) endothelial progenitor cell counts at baseline in patients with acute coronary syndrome (ACS) and patients with normal coronary arteries (normal) as assessed by coronary angiography. *p<0.05 vs normal.

**Figure B in S1 File. Relative changes in endothelial progenitor cells following treatment with reconstituted high-density lipoprotein (rHDL).** No significant changes between total number of peripheral leucocytes (CD45^+^) in patients with acute coronary syndrome following treatment with reconstituted high-density lipoprotein (rHDL) as compared to controls (Left). Relative preservation of CD34^+^ in relation to total number of peripheral leucocytes (CD45^+^) in patients with acute coronary syndrome following treatment with reconstituted high-density lipoprotein (rHDL) compared to controls (Right). Leucocytes were quantified in blood samples collected at baseline and at follow-up. The follow-up samples for the CSL-111-treated group were obtained 16 ± 4 days following completion of the 4 weekly rHDL infusions. Each box plot shows the median, the interquartile range, the maximum and the minimum of the relative change.

**Figure C in S1 File.** **Effect of CSL-111 exposure on protein expression of the receptor for SDF-1, CXCR4 in eEPCs (n=2).** Peripheral blood mononuclear cells (PBMCs) were isolated from healthy donors (n = 2) and plated on fibronectin-coated plates in the absence or presence of CSL-111 (1 mg/mL) from day 0 to day 4 (D0-4), 0 to 7 (D0-7) or 4 to 7 (D4-7). On day 7 of culture, adherent cells were harvested and proteins analysed by western blotting. The values for CXCR4 protein are reported as a ratio over the expression of GAPDH. A representative western blot is shown on top of the quantitative evaluation.

**Protocol A in S1 File. Western blot analysis of CXCR4**. RIPA-extracted proteins (50 μg/well) from eEPC exposed or not to 1 mg/mL of CSL-111 from day 0 to day 4 (D0-4), 0 to 7 (D0-7) or 4 to 7 (D4-7) were electrophoresed by SDS-PAGE and transferred to membrane. Membranes were blocked for non-specific binding in Tris-buffered saline, 0.1% Tween20 (TBST) containing 5% (w/v) non-fat milk at room temperature for 2 hrs followed by overnight incubation with anti-rabbit polyclonal CXCR4 antibody (1:2500 dilution; ab2074; Abcam) at 4°C in 5% non-fat milk. After three successive washes with TBST buffer, membrane blots were incubated with goat anti-rabbit secondary antibody (1:10000) coupled with horseradish peroxidase for 90 mins and washed again twice 10 minutes each. Protein bands were detected by ECL chemiluminescence (Pierce). Band intensities were quantified using Phoretix 1D software (TotalLab, UK). For normalization, membranes were stripped with 0.2 M NaOH and were reprobed with primary antibody to GAPDH (ab9485, Abcam) followed by secondary antibody, detected by ECL and analyzed as above.
