# Supplementary figures and images for: Beneficial Effects of Reconstituted High-Density Lipoprotein (rHDL) on Circulating CD34+ Cells in Patients after an Acute Coronary Syndrome
Source: PLoS One. 2017 Jan 6;12(1):e0168448. doi: 10.1371/journal.pone.0168448 (PMC5218493; doi:10.1371/journal.pone.0168448)

## Slide 1
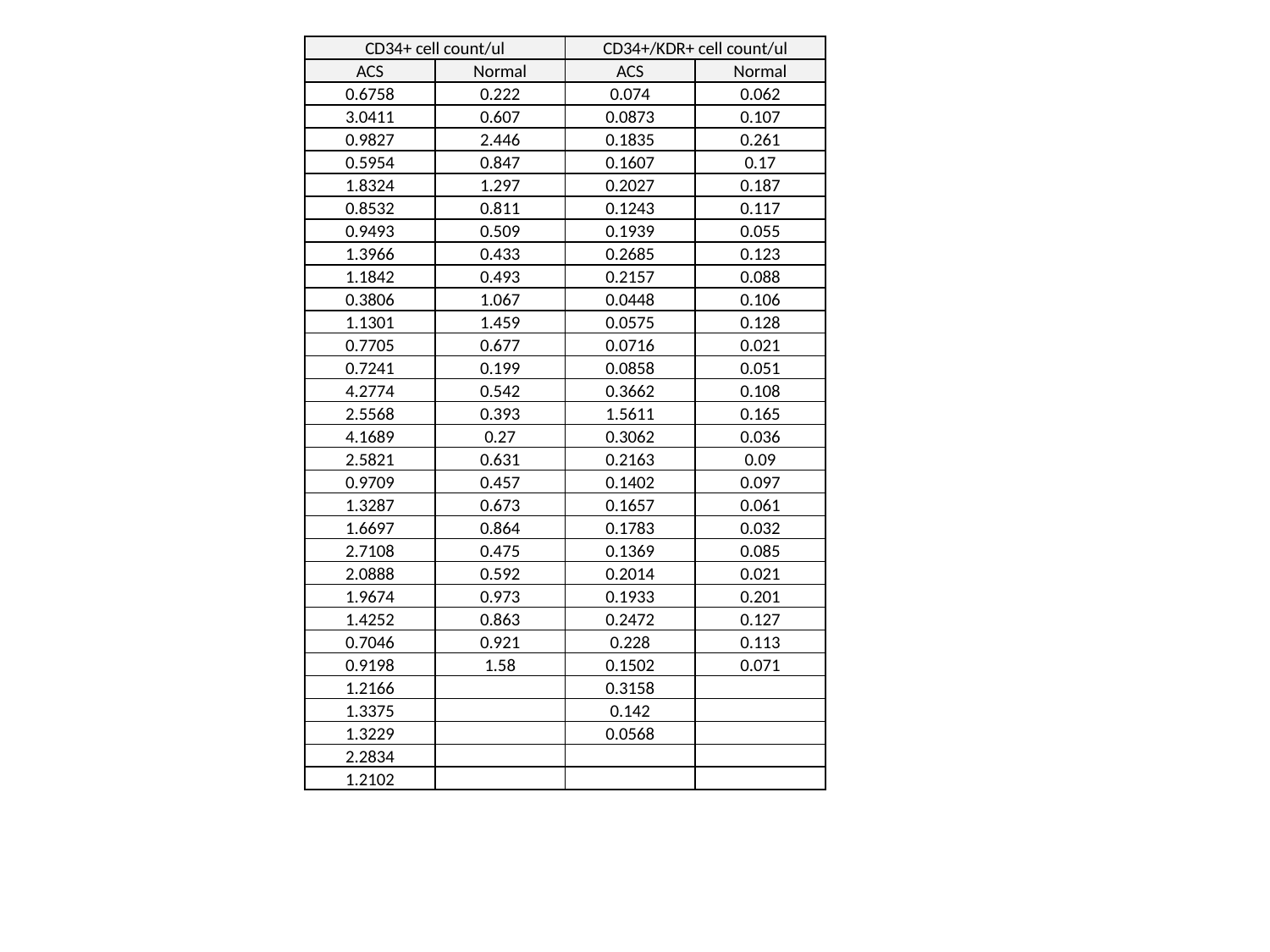

| CD34+ cell count/ul | | CD34+/KDR+ cell count/ul | |
| --- | --- | --- | --- |
| ACS | Normal | ACS | Normal |
| 0.6758 | 0.222 | 0.074 | 0.062 |
| 3.0411 | 0.607 | 0.0873 | 0.107 |
| 0.9827 | 2.446 | 0.1835 | 0.261 |
| 0.5954 | 0.847 | 0.1607 | 0.17 |
| 1.8324 | 1.297 | 0.2027 | 0.187 |
| 0.8532 | 0.811 | 0.1243 | 0.117 |
| 0.9493 | 0.509 | 0.1939 | 0.055 |
| 1.3966 | 0.433 | 0.2685 | 0.123 |
| 1.1842 | 0.493 | 0.2157 | 0.088 |
| 0.3806 | 1.067 | 0.0448 | 0.106 |
| 1.1301 | 1.459 | 0.0575 | 0.128 |
| 0.7705 | 0.677 | 0.0716 | 0.021 |
| 0.7241 | 0.199 | 0.0858 | 0.051 |
| 4.2774 | 0.542 | 0.3662 | 0.108 |
| 2.5568 | 0.393 | 1.5611 | 0.165 |
| 4.1689 | 0.27 | 0.3062 | 0.036 |
| 2.5821 | 0.631 | 0.2163 | 0.09 |
| 0.9709 | 0.457 | 0.1402 | 0.097 |
| 1.3287 | 0.673 | 0.1657 | 0.061 |
| 1.6697 | 0.864 | 0.1783 | 0.032 |
| 2.7108 | 0.475 | 0.1369 | 0.085 |
| 2.0888 | 0.592 | 0.2014 | 0.021 |
| 1.9674 | 0.973 | 0.1933 | 0.201 |
| 1.4252 | 0.863 | 0.2472 | 0.127 |
| 0.7046 | 0.921 | 0.228 | 0.113 |
| 0.9198 | 1.58 | 0.1502 | 0.071 |
| 1.2166 | | 0.3158 | |
| 1.3375 | | 0.142 | |
| 1.3229 | | 0.0568 | |
| 2.2834 | | | |
| 1.2102 | | | |

Supplement: S1 Table — CD34+ (left) and CD34+/KDR+ (right) endothelial progenitor cell counts at baseline in patients with ACS and patients with normal coronary arteries (normal) as assessed by coronary angiography. (PPTX) [file pone.0168448.s002.pptx]

## Slide 1
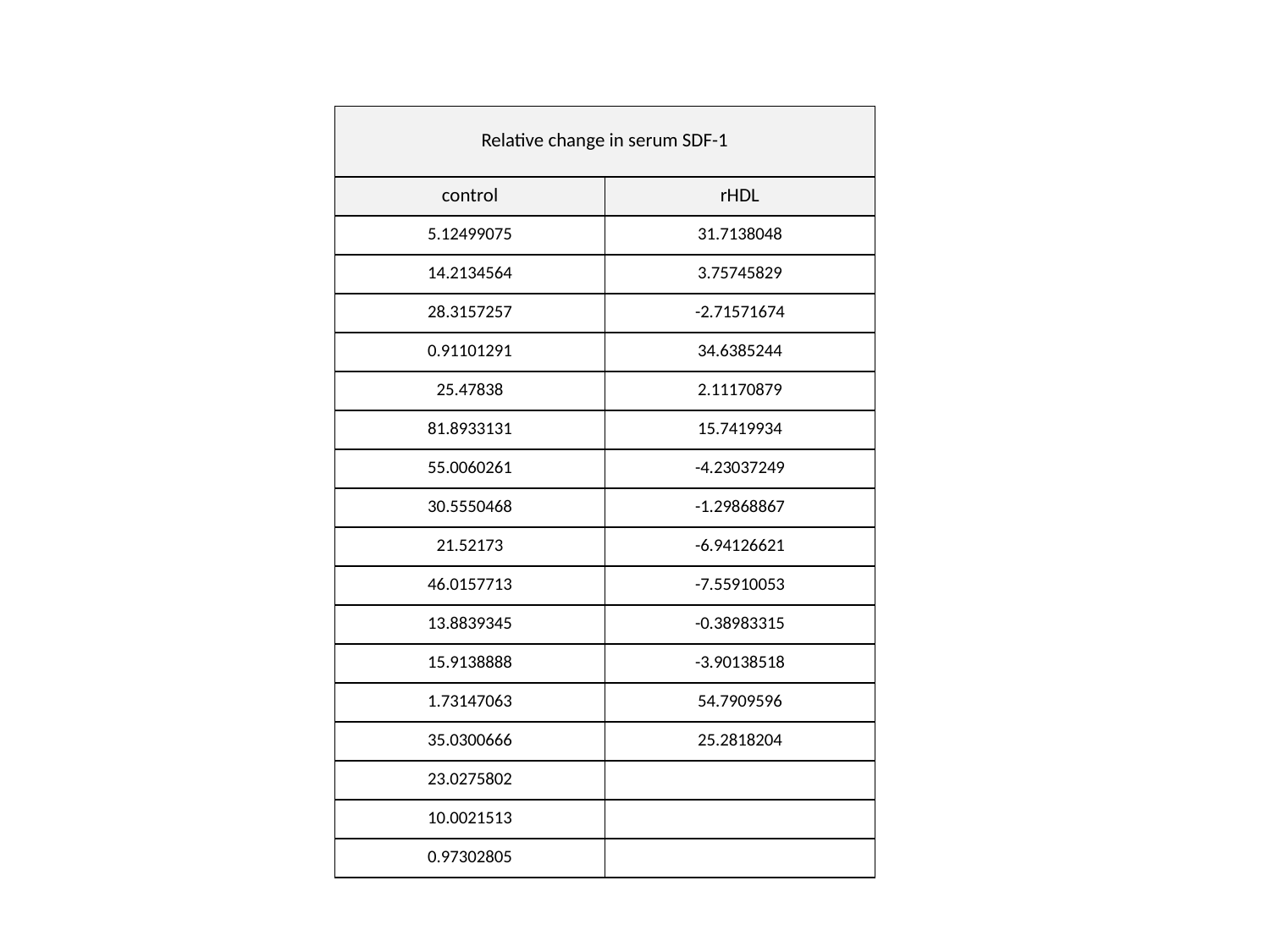

| Relative change in serum SDF-1 | |
| --- | --- |
| control | rHDL |
| 5.12499075 | 31.7138048 |
| 14.2134564 | 3.75745829 |
| 28.3157257 | -2.71571674 |
| 0.91101291 | 34.6385244 |
| 25.47838 | 2.11170879 |
| 81.8933131 | 15.7419934 |
| 55.0060261 | -4.23037249 |
| 30.5550468 | -1.29868867 |
| 21.52173 | -6.94126621 |
| 46.0157713 | -7.55910053 |
| 13.8839345 | -0.38983315 |
| 15.9138888 | -3.90138518 |
| 1.73147063 | 54.7909596 |
| 35.0300666 | 25.2818204 |
| 23.0275802 | |
| 10.0021513 | |
| 0.97302805 | |

Supplement: S3 Table — (PPTX) [file pone.0168448.s004.pptx]
