# Supplementary material for: Beneficial Effects of Reconstituted High-Density Lipoprotein (rHDL) on Circulating CD34+ Cells in Patients after an Acute Coronary Syndrome
Source: PLoS One. 2017 Jan 6;12(1):e0168448. doi: 10.1371/journal.pone.0168448 (PMC5218493; doi:10.1371/journal.pone.0168448)
Supplement: S2 Table — Left panel: CD34+ progenitor cells before and after treatment with reconstituted high-density lipoprotein (rHDL) compared to controls (Placebo treatment). Right panel: CD34+/KDR+ endothelial progenitor cells before and after treatment with reconstituted high-density lipoprotein (rHDL) compared to controls (Placebo treatment). (PPTX) [file pone.0168448.s003.pptx]

## Slide 1
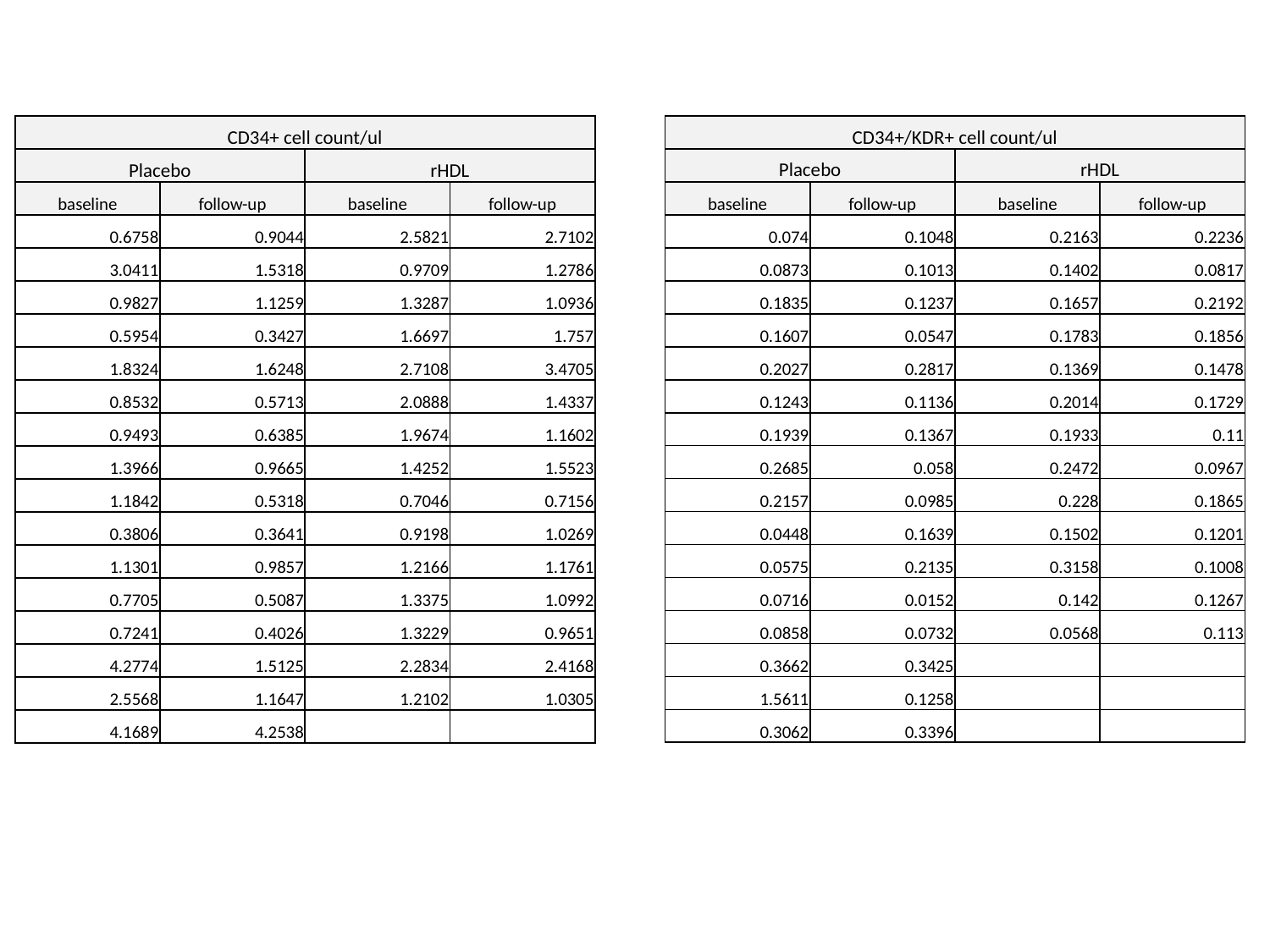

| CD34+/KDR+ cell count/ul | | | |
| --- | --- | --- | --- |
| Placebo | | rHDL | |
| baseline | follow-up | baseline | follow-up |
| 0.074 | 0.1048 | 0.2163 | 0.2236 |
| 0.0873 | 0.1013 | 0.1402 | 0.0817 |
| 0.1835 | 0.1237 | 0.1657 | 0.2192 |
| 0.1607 | 0.0547 | 0.1783 | 0.1856 |
| 0.2027 | 0.2817 | 0.1369 | 0.1478 |
| 0.1243 | 0.1136 | 0.2014 | 0.1729 |
| 0.1939 | 0.1367 | 0.1933 | 0.11 |
| 0.2685 | 0.058 | 0.2472 | 0.0967 |
| 0.2157 | 0.0985 | 0.228 | 0.1865 |
| 0.0448 | 0.1639 | 0.1502 | 0.1201 |
| 0.0575 | 0.2135 | 0.3158 | 0.1008 |
| 0.0716 | 0.0152 | 0.142 | 0.1267 |
| 0.0858 | 0.0732 | 0.0568 | 0.113 |
| 0.3662 | 0.3425 | | |
| 1.5611 | 0.1258 | | |
| 0.3062 | 0.3396 | | |
| CD34+ cell count/ul | | | |
| --- | --- | --- | --- |
| Placebo | | rHDL | |
| baseline | follow-up | baseline | follow-up |
| 0.6758 | 0.9044 | 2.5821 | 2.7102 |
| 3.0411 | 1.5318 | 0.9709 | 1.2786 |
| 0.9827 | 1.1259 | 1.3287 | 1.0936 |
| 0.5954 | 0.3427 | 1.6697 | 1.757 |
| 1.8324 | 1.6248 | 2.7108 | 3.4705 |
| 0.8532 | 0.5713 | 2.0888 | 1.4337 |
| 0.9493 | 0.6385 | 1.9674 | 1.1602 |
| 1.3966 | 0.9665 | 1.4252 | 1.5523 |
| 1.1842 | 0.5318 | 0.7046 | 0.7156 |
| 0.3806 | 0.3641 | 0.9198 | 1.0269 |
| 1.1301 | 0.9857 | 1.2166 | 1.1761 |
| 0.7705 | 0.5087 | 1.3375 | 1.0992 |
| 0.7241 | 0.4026 | 1.3229 | 0.9651 |
| 4.2774 | 1.5125 | 2.2834 | 2.4168 |
| 2.5568 | 1.1647 | 1.2102 | 1.0305 |
| 4.1689 | 4.2538 | | |
