# Supplementary material for: Beneficial Effects of Reconstituted High-Density Lipoprotein (rHDL) on Circulating CD34+ Cells in Patients after an Acute Coronary Syndrome
Source: PLoS One. 2017 Jan 6;12(1):e0168448. doi: 10.1371/journal.pone.0168448 (PMC5218493; doi:10.1371/journal.pone.0168448)
Supplement: S4 Table — Total adherent (left), CD34+(middle) and apoptotic (right) cell count in the absence (control) or presence of CSL-111 (1 mg/mL). D, day. (PPTX) [file pone.0168448.s005.pptx]

## Slide 1
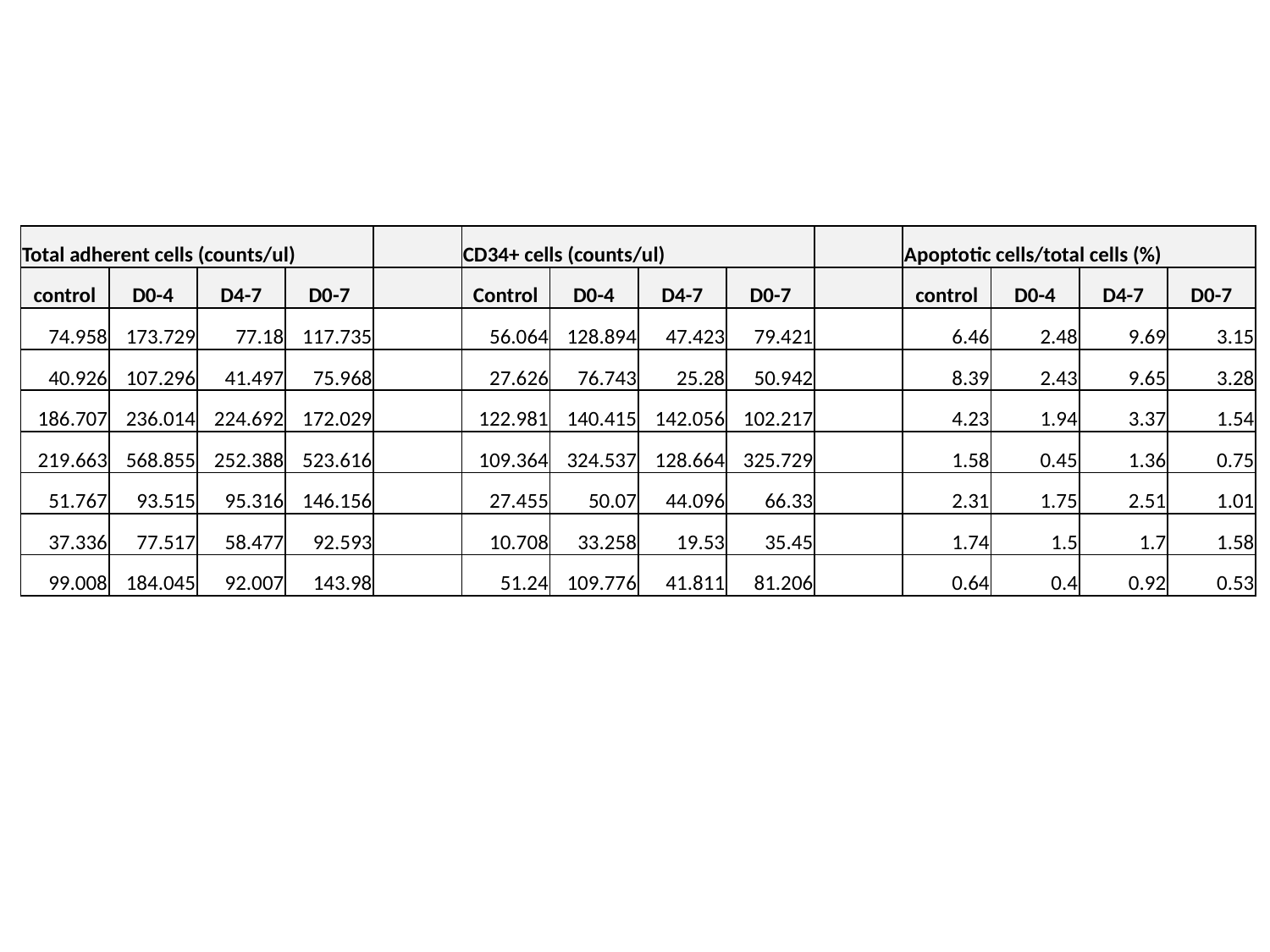

| Total adherent cells (counts/ul) | | | | | CD34+ cells (counts/ul) | | | | | Apoptotic cells/total cells (%) | | | |
| --- | --- | --- | --- | --- | --- | --- | --- | --- | --- | --- | --- | --- | --- |
| control | D0-4 | D4-7 | D0-7 | | Control | D0-4 | D4-7 | D0-7 | | control | D0-4 | D4-7 | D0-7 |
| 74.958 | 173.729 | 77.18 | 117.735 | | 56.064 | 128.894 | 47.423 | 79.421 | | 6.46 | 2.48 | 9.69 | 3.15 |
| 40.926 | 107.296 | 41.497 | 75.968 | | 27.626 | 76.743 | 25.28 | 50.942 | | 8.39 | 2.43 | 9.65 | 3.28 |
| 186.707 | 236.014 | 224.692 | 172.029 | | 122.981 | 140.415 | 142.056 | 102.217 | | 4.23 | 1.94 | 3.37 | 1.54 |
| 219.663 | 568.855 | 252.388 | 523.616 | | 109.364 | 324.537 | 128.664 | 325.729 | | 1.58 | 0.45 | 1.36 | 0.75 |
| 51.767 | 93.515 | 95.316 | 146.156 | | 27.455 | 50.07 | 44.096 | 66.33 | | 2.31 | 1.75 | 2.51 | 1.01 |
| 37.336 | 77.517 | 58.477 | 92.593 | | 10.708 | 33.258 | 19.53 | 35.45 | | 1.74 | 1.5 | 1.7 | 1.58 |
| 99.008 | 184.045 | 92.007 | 143.98 | | 51.24 | 109.776 | 41.811 | 81.206 | | 0.64 | 0.4 | 0.92 | 0.53 |
