# Supplementary material for: Beneficial Effects of Reconstituted High-Density Lipoprotein (rHDL) on Circulating CD34+ Cells in Patients after an Acute Coronary Syndrome
Source: PLoS One. 2017 Jan 6;12(1):e0168448. doi: 10.1371/journal.pone.0168448 (PMC5218493; doi:10.1371/journal.pone.0168448)
Supplement: S5 Table — Chemotactic index of endothelial progenitor cells indicating their capacity to migrate along an SDF-1 gradient following treatment with CSL-111 for different periods of time. (PPTX) [file pone.0168448.s006.pptx]

## Slide 1
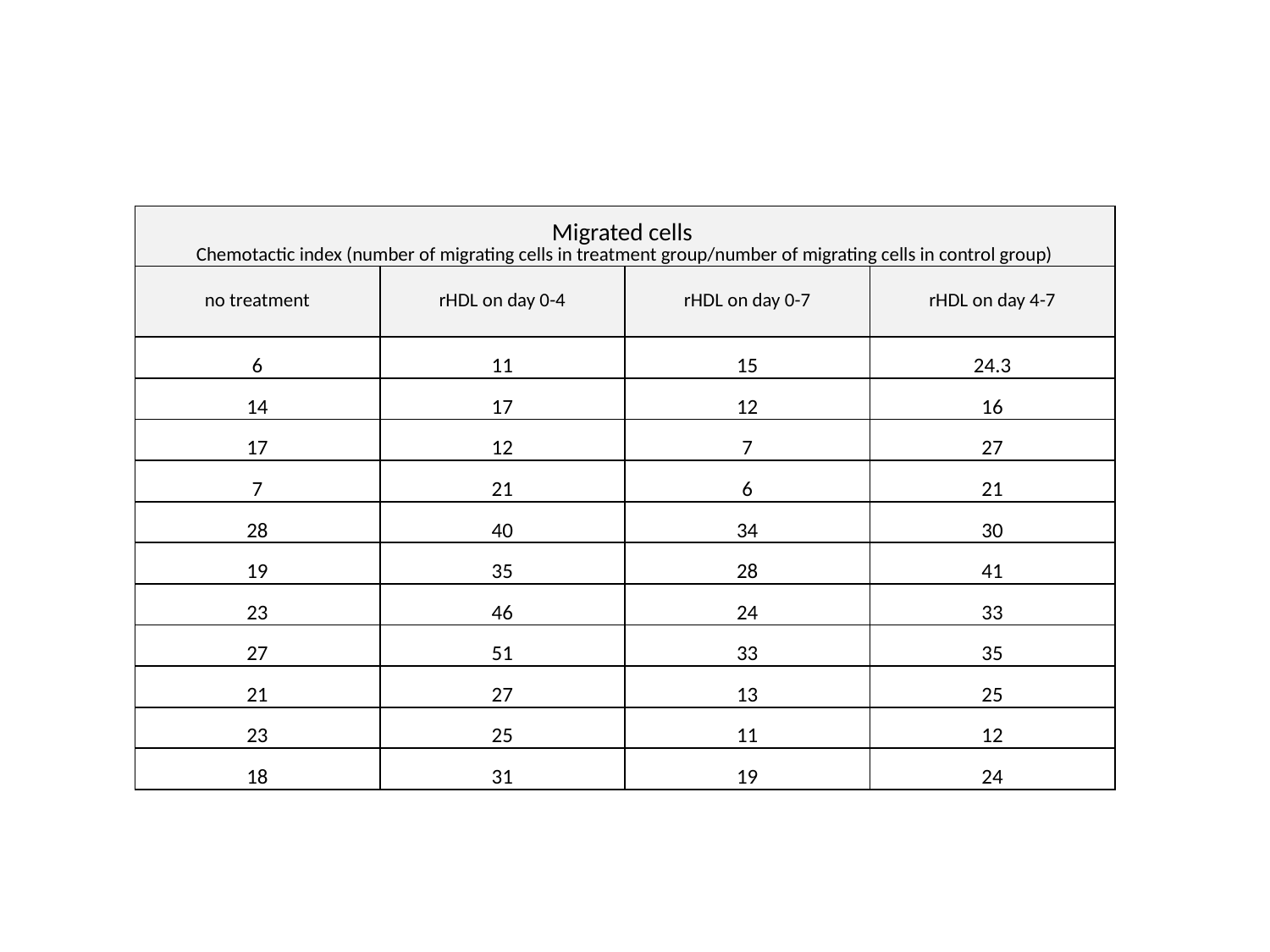

| Migrated cells Chemotactic index (number of migrating cells in treatment group/number of migrating cells in control group) | | | |
| --- | --- | --- | --- |
| no treatment | rHDL on day 0-4 | rHDL on day 0-7 | rHDL on day 4-7 |
| 6 | 11 | 15 | 24.3 |
| 14 | 17 | 12 | 16 |
| 17 | 12 | 7 | 27 |
| 7 | 21 | 6 | 21 |
| 28 | 40 | 34 | 30 |
| 19 | 35 | 28 | 41 |
| 23 | 46 | 24 | 33 |
| 27 | 51 | 33 | 35 |
| 21 | 27 | 13 | 25 |
| 23 | 25 | 11 | 12 |
| 18 | 31 | 19 | 24 |
